# From Genome-Wide to Environment-Wide: Capturing the Environome

**DOI:** 10.1177/1745691620979803

**Published:** 2021-03-01

**Authors:** Sophie von Stumm, Katrina d’Apice

**Affiliations:** Department of Education, University of York

**Keywords:** genomics, genetics, environment, large data, effect sizes

## Abstract

Genome-wide association (GWA) studies have shown that genetic influences on individual differences in affect, behavior, and cognition are driven by thousands of DNA variants, each with very small effect sizes. Here, we propose taking inspiration from GWA studies for understanding and modeling the influence of the environment on complex phenotypes. We argue that the availability of DNA microarrays in genetic research is comparable with the advent of digital technologies in psychological science that enable collecting rich, naturalistic observations in real time of the environome, akin to the genome. These data can capture many thousand environmental elements, which we speculate each influence individual differences in affect, behavior, and cognition with very small effect sizes, akin to findings from GWA studies about DNA variants. We outline how the principles and mechanisms of genetic influences on psychological traits can be applied to improve the understanding and models of the environome.

The oldest and perhaps most difficult question in psychological science is how and why people, as individuals, become who they become. There is broad consensus that people’s differences in affect, behavior, and cognition result from the interplay between genetic propensities and environmental conditions. However, the mechanisms and processes that drive this interplay are not yet well understood.

Historically, psychologists have paid greater attention to identifying the environmental influences that give rise to people’s psychological and behavioral differences than to exploring the genetic factors that might be involved. But recently, genetics has been catapulted to the forefront of psychological science because of four developments that unfolded contemporaneously in the early 2000s. First, the human genome was successfully sequenced in 2001 (Lander et al., 2001), including the identification and mapping of all genes in the human genome. The human genome is about 3 billion base pairs long, divided across 23 chromosomes, and contains around 20,000 genes. *Genes* are sequences of base pairs in the genome that code for proteins; only about 2% of the genome comprises protein-coding genes. The other 98% of the base pairs, the majority of the genome, are known as “dark matter” because much of their function is not yet well understood (Ohno, 1972).

Shortly after the completion of the Human Genome Project, the HapMap project set out to tag the DNA variants in the human genome that captured most of the genomic variation in human populations (Gibbs et al., 2003). These DNA variants are referred to as single-nucleotide polymorphisms (SNPs), which are single nucleotides that occur in different (i.e., poly) forms (i.e., morphisms) at a specific position in the genome, across coding and noncoding regions (i.e., in genes and in dark matter DNA). SNPs are the smallest units of genomic variation between any two people, and they make up about 0.2% of the base pairs in the human genome. The remaining 99.8% of human DNA is the same for all people. Completed in 2005, the HapMap project generated a map of the DNA variants in the human genome that differ between individuals (Gibbs et al., 2003). The subsequent 1000 Genomes Project then sequenced the genomes of 2,500 individuals from 26 populations and identified 85 million SNPs that differ in at least 1% of the population (1000 Genomes Project Consortium, 2015). On average, two human genomes from the same broad ancestral population, such as Europeans, differ by 4 to 5 million SNPs from one another (1000 Genomes Project Consortium, 2015).

As the methods for charting DNA advanced, commercial companies began producing data devices with the capacity to extract and store large proportions of a person’s genome (LaFramboise, 2009). These devices are known as *DNA chips* or *DNA microarrays* and can be thought of as discrete records of a person’s SNPs. Over the past 20 years, the number of SNPs that are available for analysis on common DNA microarrays has grown from 10,000 to 2.4 million (e.g., Illumina), which are judiciously selected to provide the greatest possible coverage of the genome.

The fourth development was the emergence of genomic biobanks, which store human biological material for research purposes (Hewitt & Watson, 2013). The first notable population-wide genomic biobank was authorized by the Icelandic parliament in December 1998 to include DNA samples and electronic health records of all 270,000 Icelanders (Greely, 2007). Although not without controversies and challenges, Iceland’s biobank prompted other countries to follow suit, including Estonia, Sweden, Tonga, and the Canadian province Newfoundland (Greely, 2007). One of the biggest biobanks to date, known as the U.K. Biobank, (https://www.ukbiobank.ac.uk) started in 2006 and has enrolled 500,000 volunteers ages 40 to 69 years.

Compared with the advancements in genomic research, capturing environmental factors that inform people’s psychological differences has witnessed relatively little systematic innovation and progress. This imbalance was first noted in the context of molecular epidemiology by Wild (2005), who coined the term *exposome* to describe the exposures of individuals across the life span and how those exposures relate to health (Guloksuz et al., 2018; Vrijheid, 2014; Wild, 2012). Expanding the notion of the exposome beyond disease etiology, we conceive here the *environome*, akin to the genome, that encompasses all environmental influences that give rise to people’s differences in affect, behavior, and cognition.

Relative to the research resources invested in understanding the genetic influences on psychological traits, we note first that no large-scale efforts have been completed or are even under way to comprehensively identify and document all environmental contexts that shape human life. Second, no organized map is available that charts which environmental contexts are variable across people, times, and locations, akin to the HapMap and 1000 Genomes Projects that chronicled most of the genomic variation in human populations. Third, no devices are available to comprehensively record a person’s environome so that it can be studied in and across large population samples that afford sufficient statistical power for multifactorial analyses. Although the storage, linkage, and transfer of all kinds of data have become easier and cheaper, these advances have not yet led to large, collective efforts for sampling environments, akin to the sampling of genotypes that is now routinely undertaken for biobanks.

Because the genome and the environome are fundamentally different entities, one might say that we are comparing apples with oranges. Yet apples and oranges are both fruit that are sweet; are similar in size, weight, and shape; are grown in orchards; and may be eaten, juiced, and baked (Barone, 2000). We argue here that genetic and environmental influences on people’s differences in affect, behavior, and cognition are similar enough to apply insights from one research domain to the study of the other. In other words, we propose that the recent advances in genomic research can and should be used to transform how researchers understand and model the environment beyond shaping the grasp of how DNA makes people who they are (Plomin, 2018; Visscher et al., 2017).

This article is divided into four parts. In the beginning, we review two key phenomena—polygenicity and pleiotropy—that frame the structure and functioning of genetic influences on psychological traits. In the second part, we describe what is currently known about the relation between genes and environments, specifically the mechanisms and processes that drive gene–environment interplay. In the third part, we discuss why, despite some key differences between genome and environome, effective research into one should be inspired by the other. Finally, we recommend systematic steps to bring about a comprehensive research agenda for cracking the environome so that, in the future, its understanding will rival and perhaps even exceed the current knowledge of the genome.

## Complex Peas: Polygenicity and Pleiotropy

A seminal lesson on genotype–phenotype relations involved the pea plants that the Austrian monk Gregor Mendel (1866) cultivated in his monastery’s garden during the second half of the 19th century. Mendel observed how the color of pea seeds, a discrete trait with the seeds being either green or yellow, was passed on across generations of pea plants.^1^ As a result, Mendel correctly identified the key principles of genetic inheritance, which were and continue to be fundamental to the understanding of genetics. However, the pea seeds’ color differs in two significant ways from the psychological traits that typically differentiate humans. First, most of people’s differences in affect, behavior, and cognition are influenced by both genetic and environmental factors, whereas Mendel’s pea seeds’ color varied solely as a function of genetics. Second, psychological differences are quantitative, rather than discrete or qualitative, in the way that they are normally distributed in the population. Differences in Mendel’s peas’ characteristics were caused by a single gene that was necessary and sufficient, for example, for the development of yellow or green seeds. By contrast, the genetic transmission of quantitative traits is due to many DNA variants that correlate with the variation in a trait (Fisher, 1918). This condition is known as *polygenicity*, meaning that many genetic factors influence the phenotypic expression of one trait. Polygenicity characterizes one’s psychological differences but also other quantitative phenotypes such as height, weight, and even eye and hair color.

Quantitative phenotypes that are influenced by multiple genetic variants are known as complex traits. Over the past 15 years, genome-wide association (GWA) studies have confirmed the polygenicity of many complex traits (Buniello et al., 2019; Visscher et al., 2017). GWA studies test associations between SNPs and a target phenotype, following essentially a hypothesis-free approach that is agnostic of the specific function or location or type of a SNP (Klein et al., 2005). The technical aspects of GWA studies have been discussed in detail elsewhere (e.g., Manolio et al., 2009; Visscher et al., 2017), and their statistical tools and applications are continually refined (e.g., Grotzinger et al., 2019). Relevant here is the key finding from GWA studies that genetic influences on individual differences in complex traits are due to many thousands of DNA variants that each have a very small effect size rather than to a few genes that account for a larger proportion of the variance (Chabris et al., 2015; Gratten et al., 2014). For example, for the complex trait intelligence, defined as the ability to think and reason logically, the average effect size of an associated SNP is .005% of the variance, which means that the substantial heritability of intelligence must be due to many thousands of SNPs (Plomin & von Stumm, 2018).

Just like the genetic influence on complex traits is driven by many SNPs, the environmental influence on people’s differences also arises from many factors (e.g., Boardman et al., 2013; Bradley & Corwyn, 2002; Jensen et al., 2017). Just like SNPs are spread across the entire genome, these environmental factors occur across all times, locations, and types of experiences, including—but not limited to—natural, biophysical, social, cultural, and economic environments—that is, the environome (cf. ecological systems theory; Bronfenbrenner, 1979). And just like a single SNP, a single environmental factor is unlikely to independently account for much variance in psychological differences. Consider, for example, some of the many variables that differentiate family homes: their size, the number of books available, and the ambience or comfort of the living space. If considered in isolation, each of these variables may be meaningfully associated with the psychological differences of the homes’ inhabitants. However, if entered jointly as predictors, these elements are likely to show additivity and collinearity (e.g., Tucker-Drob, 2013; von Stumm et al., 2013). That is, they will add incrementally to each other’s prediction, accounting independently for only a small percentage of variance while also sharing a substantial amount of common variance (i.e., collinearity). This lack of independence between observations applies to measures of the environome but not to SNPs, whose effects are primarily additive (except in cases of linkage disequilibrium, which are rare; Slatkin, 2008). Notwithstanding, environmental influences on psychological traits are analogous to SNP effects; that is, they are additive, at least in part, a principle that we refer to as *polyenvironicity*, for the lack of a better term.^2^

A second key finding from GWA studies is that genetic influences on complex traits are pleiotropic, meaning that a DNA variant associated with the phenotypic expression of one trait is also often implicated in the expression of other traits. That pleiotropy applies to genetic influences on related phenotypes, such as learning disabilities or psychiatric disorders, was already evident from twin studies, which compare the phenotypic resemblance of monozygotic twins, who are genetically identical, and dizygotic twins, who share on average only 50% of their segregating genes and are genetically as similar to each other as fraternal siblings (Kendler et al., 2011; Plomin & Kovas, 2005). Yet the full extent of pleiotropy across the entire genome was recognized only in the past decade, when GWA studies had become available (Cross-Disorder Group of the Psychiatric Genomics Consortium, 2013; Krapohl et al., 2016; Sivakumaran et al., 2011). Akin to our earlier comparison of polygenicity and polyenvironicity, we argue that pleiotropy applies not only to genetic influences but also to environmental influences. Thus, environments are involved in the expression of multiple psychological traits rather than exclusively affecting one.

Empirical evidence for the multitude of factors that inform environmental influence and their broad reach on psychological traits is abound (Bradley & Corwyn, 2002; Evans, 2006; Hurt & Betancourt, 2017; Taylor et al., 2017). For example, socioeconomic status (SES) contributes to child development through a myriad of pathways that often have additive effects on child outcomes (e.g., Bradley & Corwyn, 2002; Hart & Risley, 1995; Tucker-Drob, 2013). Low family SES is associated with inadequate nutrition and a lack of cognitively stimulating resources throughout childhood, coupled with wider family issues of heightened stress and negative parenting, which all impair child development. These issues exacerbate further the SES-related difficulties that emerge from living in overcrowded, insecure, and dilapidated dwellings, many times in neighborhoods in which violence is prevalent. Each of these factors in themselves may exert only a modest influence on child development, and although they share some common variance, together, they account for a considerably greater proportion of the variance in children’s outcomes than they would individually.

Analogous to genetic pleiotropy, an environmental factor that potentially affects many psychological traits is education provision. For example, Head Start, a program for providing comprehensive early childhood education, has been shown to improve cognitive performance as well as social-emotional development and physical growth in children who enrolled in the program compared with control groups (e.g., Garces et al., 2002; McKey et al., 1985), although some of these effects reduced over time (e.g., Currie & Thomas, 1995). These findings suggest that one environmental factor can have widespread influence on affect, behavior, and cognition.

In summary, recent GWA studies have confirmed that genetic influences on psychological traits are due to many SNPs (i.e., polygenicity) that are located across the entire genome and influence multiple traits (i.e., pleiotropy). We have outlined here that the same principles of polyenvironicity and pleiotropy are likely to apply to environmental influences on psychological traits.

## Genes in Their Environments

Studies that investigate how genetic factors influence phenotypic development across environments typically differentiate gene-environment correlations (GrEs) and Gene × Environment interactions (G×Es). GrEs occur when individuals’ genotypes correspond to their environments, and they can be passive, active, and evocative (Avinun, 2020; Plomin et al., 1977). Passive GrEs are typically thought of in the context of children’s genotypes correlating with the rearing environment that their parents provide for them. This phenomenon has recently become known as *nature of nurture*, demonstrating that some of parents’ genetic effects on their children are environmentally mediated (Avinun, 2020; Kong et al., 2018; Selzam et al., 2019; Wertz et al., 2019). Active GrEs occur when individuals seek out environments that correspond to their genotypes, and evocative GrEs happen when environments seek out corresponding genotypes, for example when a teacher offers special tutoring to a child. GrEs have been demonstrated to affect all environments, ranging from the amount of breastfeeding that a child receives, to the type of school they go to, to the geographical area that people opt to live in (Abdellaoui et al., 2019; Krapohl et al., 2017; Smith-Woolley et al., 2018). People select themselves into, adapt to, and shape the environments that correspond to their genotypes. In fact, many sources of behavioral influence that are thought to be environmental are actually to some degree under genetic influence. It follows that genetic and environmental factors are not independent sources of influence (Avinun, 2020; Dick, 2011; von Stumm et al., 2020) and that genotypes, genes, and SNPs are not randomly distributed across environments, with the exception of rare, random events such as natural disasters and sudden political upheavals (Kendler et al., 1993).

Whereas GrEs indicate the extent to which genes and environments are assorted, G×Es imply that genotypes respond to environmental variation in different ways. Conley’s (2009) observation that “a gene for aggression lands you in prison if you’re from the ghetto, but in the boardroom if you’re to the manor born” (p. 238) illustrates this phenomenon. The genetic propensity for aggression helps coming out on top when competing with others: In an environment in which gang fights, knife crime, and robberies are frequent, the corresponding competitive behaviors increase the likelihood for incarceration. By contrast, when business negotiations, stock market shares, and reality TV stardom are typical environments, genetic propensities for aggression are less likely to produce behaviors that lead to imprisonment or are punishable by the law.

Although an extensive, conclusive body of empirical evidence has shown that correlations between genetic factors and environments are commonplace, G×Es remain to be reliably demonstrated (Duncan & Keller, 2011; Manuck & McCaffery, 2014; Tucker-Drob & Bates, 2016). For example, a recent large-scale study that tested GrEs and G×Es across multiple genotypes, phenotypes, and environments in the prediction of educational achievement observed systematic GrEs but concluded that the contributions of G×Es were random, weak, and negligible (Allegrini et al., 2020). Compared with detecting direct effects of genotypes on phenotypes (i.e., GrE), identifying G×Es is statistically more demanding because to be adequately powered, interaction models require much larger sample sizes than tests of direct effects (Duncan & Keller, 2011; Moffitt et al., 2006). In addition, interaction effects are not limited to two variables (i.e., genetics and one environment); they can involve an infinite number of factors, meaning that two-way interactions may actually be part of three-way interactions or four-way interactions, through to *n*-way interactions. The required sample sizes for reliably identifying such interaction effects grow immeasurably. Yet the greatest challenge for identifying G×E effects in the prediction of psychological differences is not statistical power but the fact that individuals are systematically assorted to environments rather than randomly distributed across them (Schmitz & Conley, 2017). It follows that observing truly exogenous environmental effects is likely to be impossible—except for random events that are rare (Plomin & Daniels, 1987).

## From the Genome to the Environome: Looking at the Differences

In this article, we argue that some of the principles that underlie genetic influences on psychological traits may also apply to understanding and modeling environmental influences. We hasten to acknowledge that the genome and the environome diverge fundamentally in many ways. However, we contend that overall, the similarities of genome and environome are greater than their differences, and thus, using insights from genetics to inspire a new research agenda for the environment is both justified and promising.

Stability, or lack thereof, is the first glaring difference between genome and environome. Individuals’ genomes do not change across the life span; they are formed and fixed at conception from the mother’s egg and the father’s sperm. What does change over time, however, is gene expression, a process that involves transcribing DNA into RNA and translating RNA into amino acid sequences, the building blocks of proteins. Each of these steps is regulated, largely as a function of the environmental conditions that are encountered (i.e., epigenetics). And these environmental conditions vary second by second. In contrast to the genome, the environome is pure change: No moment in people’s lives is like any other before or in the future. Notwithstanding, some environmental factors exert relatively stable influences over time, such as the characteristics of the family home that children are raised in (e.g., Bradley & Corwyn, 2002).

The second difference lies in the unit of analysis. For genetic influences, the smallest unit of variation is based on SNPs that all adhere to the same structural and functional (coding) mechanisms. Thus, an individual SNP’s effect on a complex trait is directly comparable with the effects of other SNPs (Klein et al., 2005). By contrast, the environome spans events and experiences across all perceptive senses (i.e., vision, sound, touch, taste, smell, and social perception), none of which can be traced down to core elements or analogous units of analysis. For example, one would struggle to argue that the environmental influence of the wealth of the neighborhood district on children’s cognitive growth is the same category of environmental exposure, operating through the same mechanisms, as the number of languages spoken in the family home. Statistical estimates of the effect size of both variables’ influence may help identify which one carries more weight in predicting cognitive growth. However, such estimates cannot offer a theoretical or empirical rationale for determining what constitutes a unit of environmental influence.

It is difficult, perhaps even impossible, to identify truly environmental influences, in the way that they are external to a person (Dick, 2011; Schmitz & Conley, 2017), because people are not randomly distributed across environments (i.e., the endogeneity problem). In an attempt to address the entanglement of internalized and external environments, the bioecological model of human development proposed differentiating “empirically assessable mechanisms, called *proximal processes*, through which genetic potentials for effective psychological functioning are actualized” (Bronfenbrenner & Ceci, 1994, p. 568) from the environments in which these processes operate. Proximal processes are described as “progressively more complex reciprocal interaction[s] between [a] human organism and the persons, objects, and symbols in its immediate environment” (Bronfenbrenner & Ceci, 1994, p. 572). Examples of such proximal processes include parent–child activities, reading, play, and acquiring new knowledge and know-how, all of which can be thought of as complex traits that are at least partly genetically influenced (e.g., Polderman et al., 2015; Wertz et al., 2019). In the context of the bioecological model, a child-teacher relationship may count as proximal process, whereas the wider school environment may be treated as external environment (cf. Bronfenbrenner & Morris, 2006). Although this distinction appears useful for developing a hierarchy of the propinquity of different environmental influences, it remains false: Neither teachers nor children are randomly assorted to schools (e.g., Domingue et al., 2018; Reeves et al., 2017), and thus, the wider school environment is just as external or internalized as any child–teacher relationship.

The third key distinction between genome and environome concerns their respective frameworks of transmission. For the genome, functional paths and operating mechanisms have been identified that guide the transcription and expression of genetic factors (i.e., functional genomics). Although the understanding of the corresponding processes is currently incomplete, a schematic framework exists that outlines how the genome relates to the transcriptome, proteome, and metabolome. For environmental influences, however, no systematic framework of transmission is readily available. Even for environmental factors whose influence on complex traits has been conclusively demonstrated, for example, the benefits of large quantities of child-directed speech for children’s own language development (Hart & Risley, 1995), it is unclear how they become “transcribed” to influence a phenotype. Conceiving a working framework of transmission for the environome seems an even greater challenge when one recalls the multisensory nature and function of environmental influences.

## Capturing the Environome

What, then, are the similarities between genome and environome? We argue that there is a parallel between the events that brought about the “DNA revolution” (Plomin, 2018) and those that currently inform research programs into environmental influences on individual differences in psychological traits. The advent of inexpensive DNA microarrays that made genotyping multiple regions of the genome, and even the whole genome, affordable was instrumental to the success of GWA studies. We contend that psychological science is currently witnessing the introduction of comparable technical innovations that enable capturing the environome across levels, dimensions, and time in unprecedented depth and detail.

One seminal example of an extensive, technology-driven approach from developmental research is the human speechome project. Deb Roy, professor of social machine analytics at the Massachusetts Institute of Technology, installed cameras and microphones in his house that recorded everything that happened for up to 10 hr per day—starting on the day that his son Dwayne was first brought home after his birth and lasting for the first 3 years of Dwayne’s life (Roy et al., 2009). The resulting corpus contains more than 100,000 hours of multitrack recordings that enabled Roy and his colleagues to study—in exceptional detail—the contexts in which Dwayne acquired language. Roy and his team showed that Dwayne learned words that were constrained to particular times and locations, for example mealtimes that took place in the kitchen at regular times, earlier than words that were used in multiple contexts (Roy et al., 2012). These data illustrate the importance of assessing the wider context of language acquisition, including frequency, timing, and location, to understand the underlying developmental processes. Although the corpus currently holds observations of only one child, it is easy to forecast how extending the speechome project will help to identify the environmental conditions that influence differences in the speed and proficiency with which children learn words. For example, we might hypothesize on the basis of Roy et al.’s (2012) findings that children who grow up in less contextualized homes, for example where meals are eaten at irregular times in rooms that are also frequently used for other activities, show greater delays in the acquisition of language.

Other examples of new tools that enable collecting real-world observations of individual differences in affect, cognition, and behavior in exceptional depth include technology-supported experience sampling methods (Harari et al., 2016; Wrzus & Mehl, 2015). For example, LENA is a technology that extracts the number of words that a child hears over the course of a day from digital audio recorders (d’Apice et al., 2019). Other tools such as mobile sensors, accelerometers, and pulse-oximetry-based monitors allow tracking motor activity, sleep patterns, and autonomic nervous system activities as they occur in real time (Bonafide et al., 2018; de Barbaro, 2018). Technology-supported experience sampling methods and their extensions can be further enriched by linking their data with records of the wider environment, for example the air quality and traffic density, or using geo-coding techniques that capture neighborhood and landscape features (Little et al., 2019). Most previous research has focused on testing the effect of one or two environmental influences that stemmed from one or two sources. Compare this approach with candidate-gene studies that tried to establish meaningful effects of a few specific genetic factors on phenotypic outcomes and failed spectacularly (Ioannidis, 2005). If environmental influences resemble genetic ones in the way that they are characterized by polyenvironicity and pleiotropy, they should be captured holistically across the entire environome.

## Cracking the Environome

Throughout this article, we have highlighted ways in which psychological science may take inspiration from genomic research to advance the understanding and models of environmental influences. Our aim is now to outline the steps that we believe are essential to bring about an effective research agenda for the environome.

A first challenge—having the technical tools available to capture the environome—is under way, although it is far from being complete. The environome comprises an infinite number of dynamic processes, whose assessment requires robust technologies that enable collecting precise, in-depth observations at multiple time points with little measurement error (Wild, 2012). Although assessment technologies have rapidly improved in recent years, capturing even one individual’s environome in its totality remains impossible to date (Roy et al., 2009).

The second challenge is to develop the computational methods required for modeling these rich data, for example using machine-learning approaches such as data mining and cluster analysis. This challenge is not specific to studies of the environome but shared with analyses of the genome. Although current GWA studies already incorporate a vast number of SNPs, they typically include only a fraction of the potentially available genomic information (Wainschtein et al., 2019). Another parallel between genome and environome suggests itself here: GWA studies currently consider only additive effects of SNPs, although interactions are plausible. Likewise, environmental factors are likely to involve interactive effects between each other in addition to additivity and collinearity. We predict that statistical advances in genomics will prevail at a fast pace and that they will be applicable not only to the genome but also to studies of the environome.

The third challenge is to develop a theoretical framework for organizing and modeling the environome and its influence on complex traits. We anticipate that this challenge can be met only through large-scale collaborations, akin to the consortia that dominate contemporary genetic research, such as the Psychiatric Genomics Consortium; https://www.med.unc.edu/pgc/) that focuses on mental health issues or the Social Science Genetic Association Consortium (https://www.thessgac.org/) that targets social science outcomes, as its name suggests. These and other consortia like them typically involve hundreds of researchers and organizations that engage in interdisciplinary collaborations and pool data across biobanks, population cohort studies, and independent samples. They offer extraordinary opportunities for scientific breakthroughs: The majority of the recent discoveries about the role of genetic influences of people’s differences in psychological traits emerged on the back of the work completed in consortia. For modeling the environome, longitudinal population cohort studies, which are typically defined by the year or decade of the cohort members’ birth and by the geographical scope from which they were recruited, will be of particular value (Cave & von Stumm, 2020). For one, longitudinal cohort studies can elucidate at least some of the environome’s dynamic changes that occur across people’s life span because cohort members are repeatedly assessed over time, including observations of the prenatal environment in some cases. For the other, population cohort studies are key to exploring the environome’s socio-historical development across generations—in other words, how the environmental experiences of today’s children differ from their parents’ and grandparents’ environmental experiences.

Rather than creating new consortia or shifting attention away from existing ones, we suggest broadening their scope to also pool data and expertise on the environome. Akin to the HapMap Project, a first step for a systematic research program into the environome would call for charting the breadth of environments that humans experience. A bottom-up approach, for example by creating comprehensive archives of environmental measures that are available across biobanks, population cohort studies, and independent samples, has some appeal. The alternative top-down approach would involve developing a theoretical taxonomy that could be applied to categorize observations of environments, including those already collected in previous studies, and then be subjected to empirical validation. An encouraging example is the DIAMONDS taxonomy that proposes eight dimensions to classify psychological situations by the extent to which they pertain to **d**uty (i.e., something has to be done), **i**ntellect (i.e., learning opportunity), **a**dversity (i.e., threat), **m**ating (i.e., sexually charged), p**o**sitivity (i.e., playfulness), **n**egativity (i.e., stress), **d**eception (i.e., sabotage), and **s**ociality (i.e., social interaction; Rauthmann et al., 2014). Although the DIAMONDS taxonomy has to date been applied to only a select number of contexts and is fairly abstract, its theoretical framework may inspire analogous models for describing the environome.

GWA studies serve to identify genetic predictors of developmental differences in psychological traits, but they currently offer little value for elucidating the causality that underlies this prediction (Belsky & Harden, 2019). Likewise, the framework we proposed here for modeling the environome focuses on prediction. It does not qualify for finding the functional or causal mechanisms that explain why certain environmental conditions benefit phenotypic development more than others. Although not always appreciated, accurate prediction of psychological traits is immensely precious in itself because it enables identifying risk and resilience before problems manifest. In addition, a better understanding of the environome will help generate hypotheses that in the future can facilitate direct tests of causality, akin to current endeavors in functional genomics that try to make sense of gene and protein functions and interactions.

## Transparency

*Action Editor:* Laura A. King

*Editor:* Laura A. King

## Notes

1. Mendel (1866) worked overall with seven discrete characteristics of pea plants: seed shape and color, pod shape and color, flower position and color, and plant height. His findings were consistent across all seven pea plant characteristics.

2. In writing this article, we could not help but notice the paucity of words available to describe environmental influences. This contrasts with a relative richness and precision of terminology surrounding genetic factors, for example *DNA*, *DNA variants*, *SNPs*, *heritability*, *genes*, and the more flowery *language of life*.
